# Effects of *Angelica archangelica* Extract on Overactive Bladder: A Pilot Randomized Controlled Trial

**DOI:** 10.1002/fsn3.71258

**Published:** 2025-12-08

**Authors:** Jaime López‐Seoane, Eva Gesteiro, María José Castro‐Alija, Carlos Quesada‐González, Margarita Pérez‐Ruiz, Marcela González‐Gross

**Affiliations:** ^1^ ImFINE Research Group, Department of Health and Human Performance, Faculty of Physical Activity and Sport Sciences‐INEF Universidad Politécnica de Madrid Madrid Spain; ^2^ Red Española de Investigación en Ejercicio Físico y Salud (EXERNET) Zaragoza Spain; ^3^ Research Group on Assessment and Multidisciplinary Intervention in Health Care and Sustainable Lifestyles, Faculty of Nursing University of Valladolid Valladolid Spain; ^4^ Department of Mathematics Applied to Information and Communication Technologies Universidad Politécnica de Madrid Madrid Spain; ^5^ Department of Biomedical Research Center of Pathophysiology of Obesity and Nutrition‐CIBERobn, Carlos III Health Institute Madrid Spain

**Keywords:** clinical practice, health, natural supplementation, nocturia, overactive bladder, plant products, traditional medicine

## Abstract

Overactive bladder (OAB) is a prevalent syndrome affecting quality of life (QoL) in 10.8% of men and 12.8% of women in Europe, impacting various aspects of daily functioning. Conventional treatments like antimuscarinic drugs show low adherence due to limited efficacy and side effects. This study explores the effects of natural supplementation with *Angelica archangelica* (AA) leaf extract on OAB symptoms, focusing on daytime voids, nocturia, and urgency. A pilot study with a randomized, triple‐blind, controlled trial design, registered on ClinicalTrials.gov (NCT04357223), was conducted with 143 participants assigned to AA (SUP) or placebo (PLA) groups in a 1.75:1 ratio over 6 weeks. Adults aged 18–75 with specific OAB symptoms were included. Data were securely managed via the RedCap platform. OAB symptomatology was assessed following the recommended tools and scales according to the Spanish Association of Urology guidelines: A 3‐day voiding diary (3‐dVD) and the International Prostate Symptom Score (IPSS). Additionally, the IPSS total score and IPSS storage subscore were measured. Statistical analysis was performed by SPSS, R, and Python. The Wilcoxon test was used to analyze pre‐to‐post differences, and linear mixed model analysis for group‐by‐time interactions. SUP showed significant improvements in daytime voids (*p* = 0.004; η^2^_g_ = 0.07), IPSS storage subscore (*p* = 0.025; η^2^_g_ = 0.05), and QoL (*p* < 0.001; η^2^_g_ = 0.12), while a nearly significant improvement in nocturia was observed through IPSS results (*p* = 0.069; η^2^_g_ = 0.03) compared with PLA. IPSS total score changes were clinically meaningful and higher in SUP (from 19.78 to 15.89) than in PLA (from 19.64 to 16.38). Adults with OAB syndrome significantly reduced daytime voids and IPSS storage subscore, improving QoL after AA supplementation for 6 weeks. Positive effects on nocturia were also observed.

**Trial Registration:** ClinicalTrials.gov identifier: NCT04357223

## Graphical abstract

Overactive bladder (OAB) affects 10.8% of men and 12.8% of women in Europe, significantly impairing quality of life (QoL). This randomized, triple‐blind, controlled pilot trial evaluated *Angelica archangelica* (AA) leaf extract versus placebo in 143 adults with OAB over 6 weeks. Outcomes were measured using a voiding diary and IPSS. Results showed significant improvements in daytime voids, IPSS storage subscore, and QoL, with favorable trends in nocturia. IPSS total score changes were clinically meaningful and higher in SUP than PLA. No significant changes in urgency were observed.

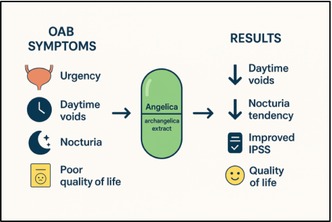

## 1 Introduction

The overactive bladder (OAB) syndrome is a prevalent condition affecting 10.8% of men and 12.8% of women in Europe (Cheng et al. 2024; Eduardo Martínez et al. 2009; Stewart et al. 2003). It adversely affects the quality of life (QoL) of patients by impacting social, sexual, and work‐related functions. Consequently, OAB imposes a significant issue not only on patients' daily life but also on public health systems and society (Sacco et al. 2010). OAB's origin is idiopathic, needing the exclusion of other conditions such as urinary infections, bladder tumors, lower urinary tract obstruction, or neurological disorders before diagnosis. OAB is characterized by the presence of urgency to urinate, with or without incontinence, often associated with polyuria and nocturia (Abrams et al. 2002; Haylen et al. 2010).

Currently, pharmacological treatment includes antimuscarinic drugs, although it shows low adherence among OAB patients due to the perceived lack of efficacy and the associated adverse effects such as dizziness or urinary discomfort (Benner et al. 2010; Wee et al. 2025; Vouri et al. 2017). Therefore, it is imperative to explore effective alternative treatments to alleviate OAB symptoms and improve QoL. A combination of treatment strategies may be the most effective approach for symptom relief in OAB patients. In this regard, natural supplementation could provide benefits on the OAB symptomatology, avoiding the adverse effects of the pharmacological treatment.

*Angelica archangelica* (AA) belongs to the *Apiaceae* family. It is native to Western Asia, northern Europe, Scotland, and Syria, and has a rich history both in folk medicine and as a food source. Different studies show that extracts from its roots, fruits, leaves, and whole plant contain various bioactive compounds with significant biological effects (Kaur and Bhatti 2021; Patyra et al. 2024). In the context of OAB, some of these components have been proposed to modulate processes associated with smooth muscle contractions (Sigurdsson et al. 2013), the regulation of neurotransmitters involved in the neural innervation of bladder muscles (Gasmi et al. 2022), anti‐inflammatory activity and inhibition of leukotriene (LTD4) activity which potentially reduced bladder inflammation (Batiha et al. 2022; Ponce‐Monter et al. 2008; Sigurdsson et al. 2013; Yoshimura and Chancellor 2001), and antioxidant properties that could protect bladder cells from oxidative damage (Batiha et al. 2022). All these mechanisms could contribute to symptom relief through different pathways; however, while AA may represent a possible adjunctive option for managing this condition, evidence supporting its specific benefits remains limited.

Thus, the aim of this study was to analyze the effects of natural supplementation with AA on individuals with signs, symptoms, and/or a diagnosed OAB condition.

## 2 Methodology

### 2.1 Study Design

This was a pilot study with a randomized controlled trial (RCT) design conducted in adults suffering from OAB syndrome. It was conducted with a matched pair trial design, an exploratory framework, and a 1.75:1 allocation ratio between the experimental group (SUP) and the placebo group (PLA), respectively. The OAB study was performed at the Biochemical Laboratory of the Faculty of Physical Activity and Sports Science at the Universidad Politécnica de Madrid (UPM), in Madrid, Spain. All participants were examined by the same researchers at baseline (T0) and at the end of the 6‐week supplementation period (T6).

### 2.2 Participants

#### 2.2.1 Recruitment

Volunteers older than 18 years, including both men and women, with signs, symptoms, and/or diagnosed OAB, were recruited by snowball sampling. Social media, digital display screens in public areas of the UPM, e‐mail campaigns, and the ImFINE Research Group (ImFINE‐RG) local online contact website (https://imfine.com.es/vejiga‐hiperactiva/) were used to reach adults interested in participating in the OAB study. Participants initially completed and signed the Informed Consent Form. Then, potential participants were contacted through phone calls to provide a broader overview of the trial. Those who volunteered for the study were registered, assigned to an anonymous participant code (see the “Data storage” section), and filled in the Inclusion and Exclusion Criteria Form. After completing these initial questionnaires, participants were enrolled, and their data were checked for eligibility by a researcher. Participants did not receive any financial compensation for volunteering in this study, but they were given personalized nutritional advice and individual reports of all clinical and physical measurements performed at the end of the study.

The Research Electronic Data Capture Platform (RedCap), hosted at the Supercomputing and Visualization Center of the Universidad Politécnica de Madrid (CESVIMA‐UPM), was used to digitally secure the field work of the study. RedCap is a specialized tool for building and managing clinical trials and their corresponding databases.

#### 2.2.2 Inclusion Criteria

The participants who accomplished the following inclusion criteria were included in the study: (i) age between 18 and 75 years; (ii) presence of OAB symptoms, as defined by the Spanish Association of Urology, with symptom assessment based on the IPSS. Inclusion criteria required meeting the following three criteria according to the IPSS: urgency to urinate (score ≥ 2 on question 4), nocturia (defined as waking up more than once per night to urinate, score ≥ 2 on question 7), and frequent urination (defined as needing to urinate again less than 2 h after the last urination, score ≥ 2 on question 2).

#### 2.2.3 Exclusion Criteria

Participants with the following conditions were excluded from the study: (i) abnormal urinary findings suggestive of urinary tract infection, significant hematuria or glucosuria requiring further evaluation; (ii) subjects who had undergone surgical treatment for bladder outlet obstruction/benign prostatic hyperplasia within the past 6 months; (iii) subjects with a history of urological malignancy (e.g., bladder cancer, prostate cancer); (iv) subjects with a medical history or active medical conditions that would prohibit participation in the study (including, but not limited to: diabetes, cancer, renal failure, cirrhosis or chronic liver disease, pancreatic disease, recent myocardial infarction (< 6 months) or unstable coronary artery disease); (v) subjects starting with medications affecting urination within the 2 months prior to randomization [e.g., loop diuretics (furosemide), antimuscarinic agents, finasteride or dutasteride]; (vi) subjects using natural products for benign prostatic hyperplasia, such as saw palmetto (*Sabal serrulata* or *Serenoa repens* ); (vii) subjects who had been using AA supplements or other products containing AA within the 2 months prior to randomization; (viii) subjects with known allergy to AA or any other ingredients of the supplement used in present research; (ix) subjects who had received an investigational product within 30 days before enrollment or expected receipt during this study; (x) and subjects whose work or lifestyle potentially interfered with regular night‐time sleep (e.g., shift workers).

#### 2.2.4 Data Storage

Complete individual records were documented using a unique, anonymous participant code and did not contain identifiable participant information. Participants' numeric codes were randomly assigned in RedCap with five‐digit numbers. Complete records for each participant were stored on the RedCap platform. Digital databases were created for each assessment and instrument, ensuring data quality and eliminating the need to duplicate questionnaire measurements. These databases were secured with a password accessible only to the study coordinator and principal investigator (PI) of the OAB study (J.L.‐S. and M.G.‐G., respectively). Additional researchers could access the password and study databases only with authorization from the PI. The PI made all final decisions regarding RedCap platform management, trial supervision, and the start and stop points of the trial.

#### 2.2.5 Sample Size

The available data regarding the effect of phytotherapeutic agents on OAB are limited. A literature review of previous pilot studies found that the mean reduction in nocturnal voids was between 1 and 2 for the interventions, while the placebo effect ranged from 0.2 to 0.8 (Andersson and Van Kerrebroeck 2018; Han et al. 2017; Sakalis et al. 2017; Weiss and Everaert 2019). Thus, it was calculated that a total of 138 participants (79 in SUP and 59 in PLA) should provide a probability of at least 80% that the study will detect a difference between the supplement and placebo at a two‐sided 5% significance level, if the true difference between the groups is around 0.7 nocturnal voids and the standard deviation is 1.0 (Fu et al. 2011).

#### 2.2.6 Randomization

For randomization, a researcher not involved in the study used individual codes and the randomization function of Microsoft Excel. Considering the high experimental dropout rate in studies related to lower urinary tract diseases (Chin et al. 2017; Ferry et al. 2004), the randomization was set at 1.75:1 to ensure the representativeness of the SUP. Then, the 143 participants included in the OAB study were finally randomly assigned to SUP (*n* = 84) and PLA (*n* = 59).

#### 2.2.7 Blinding

Triple‐blinding allowed neither the participants nor the PI nor the data analyst to know each participant's assignment.

#### 2.2.8 Supplement Intervention

The 6‐week intervention of the OAB study consisted of taking a supplement with AA leaf extract during 6 weeks for participants belonging to SUP, and taking a placebo during 6 weeks for participants belonging to PLA. Subjects in SUP consumed two capsules a day of SagaPro (Florealis ehf, ISL), the daily dose being 200 mg AA leaf extract. Subjects in PLA consumed two capsules a day composed only of the supplement excipient (Florealis ehf, ISL). The capsules of each group were identical in appearance and flavor. The 84 capsules were provided in a gray can labeled only with the code of each participant. To ensure compliance, participants were required to return the empty gray can at the end of the intervention. Participants were asked to maintain their habitual diet, physical activity, and exercise during the intervention. To monitor this, a telephone call was made at week 3 of the intervention to ensure compliance and, during the two visits (T0 and T6), participants completed a 24‐h dietary recall (Salvador Castell et al. 2015) (data not included) and the International Physical Activity Questionnaire Long Form Spanish version (Craig et al. 2003) (data not included).

#### 2.2.9 Ethical considerations and clinical trial registration

This research was performed in accordance with the Ethical Guidelines of the Declaration of Helsinki of 1964 and further amendments (“Declaration of Helsinki. Recommendations Guiding Doctors in Clinical Research.,” 1964; World Medical Association 2013). The protocol was approved by the Ethics Committee of the UPM (reference number 20200305–1, date: 16/03/2020) and registered on ClinicalTrials.gov (Clinical Trials ID NCT04357223, name: “NOGO for an Overactive Bladder”). Written informed consent was obtained from each participant before the start of their participation and after explaining the aims, risks, and potential discomfort associated with the study. This study was performed by qualified professionals for medical, clinical, nutritional, and Physical Activity and Sport Sciences practice of the ImFINE‐RG of the Department of Health and Human Performance of the Faculty of Physical Activity and Sport Sciences of the UPM.

### 2.3 Outcomes

#### 2.3.1 Anthropometric Variables

Age and sex were recorded at the study outset. Height (cm) was measured using a 2‐m stadiometer (Seca 213, Hamburg, Germany) in a standing position and vertical plane. Weight (kg) and body composition data (total fat mass (% and kg), total fat‐free mass (kg), body mass index (BMI) (kg/m^2^), and basal metabolic rate (BMR) (kcal)) were assessed by a bioelectrical impedance analysis (BIA) (MC‐780 MA, Tanita, Tokyo, Japan).

#### 2.3.2 OAB Outcomes

OAB symptomatology was assessed through a 3‐day voiding diary (3‐dVD) (Adot Zurbano et al. 2015; Jimenez‐Cidre et al. 2015) and the International Prostate Symptom Score (IPSS) (Badía et al. 1998; Barry et al. 1992) at T0 and T6. These questionnaires are among the recommended tools and assessment scales for OAB symptoms according to the 2019 guidelines on OAB by the Spanish Association of Urology (Adot Zurbano et al. 2015).

##### 2.3.2.1 3‐dVD

Participants were instructed to fill out the 3‐dVD correctly and completely during T0. The participants recorded, on the sheet for each day, the time they got up and went to bed and the time they urinated, the urgency intensity according to the Patient Perception of Intensity of Urgency Scale (PPIUS), and registered if they had any leaks, the need to change (e.g., underwear, use of pads), and fluid intake. The amount of urine was expressed in mL and measured with a plastic measuring jar. Daytime voids were measured by assessing the number of voids while participants were awake, and nocturia was considered as the number of times participants woke up to urinate while sleeping at night. Urgency was classified in five degrees according to the PPIUS (0 = no urgency; 1 = mild urgency; 2 = moderate urgency; 3 = severe urgency; and 4 = urgency incontinence).

##### 2.3.2.2 IPSS

Regarding the IPSS questionnaire, it consists of eight questions that are used to assess voiding symptoms (incomplete emptying of the bladder (Q1), intermittency (Q3), weak stream (Q5), and straining to void (Q6)), storage symptoms (daytime voids (Q2), urgency (Q4), and nocturia (Q7)) and QoL (Q8) (Barry et al. 1992; Liao and Kuo 2014). Questions Q2, Q4, and Q7, as well as the measurement of QoL (Q8), were analyzed separately, and the IPSS was also divided into the total score and the storage subscore (Liao and Kuo 2014; Shalaby et al. 2013). Changes of more than three points in the IPSS total score were considered clinically meaningful (Barry et al. 1995; Hollingsworth and Wilt 2014).

### 2.4 Statistical Analysis

Data analyses were performed with IBM SPSS for Windows v29.0 (IBM Corporation, Armonk, NY, USA), R version 4.3.0 under the frontend RStudio2023.06 and Python 3.13. The values shown for quantitative and ordinal variables were the mean ± standard deviation (SD). Results were analyzed per protocol. The assumption of normality was tested with the Kolmogorov–Smirnov test and the assumption of homogeneity of variances with the Levene test. The Mann–Whitney U test was used to analyze group differences at T0. The Wilcoxon test was performed to analyze time interactions in SUP and PLA independently. To determine the group‐by‐time interaction on the IPSS scores and the 3‐dVD results, a linear mixed model was carried out, and the interaction term is reported. As an index of effect size, η^2^_g_ was used, following the interpretation by Cohen (1988); small effect sizes from ≥ 0.01 to < 0.06, medium from ≥ 0.06 to < 0.14, and large ≥ 0.14. The significance level for all procedures was set at 0.05.

## 3 Results

### 3.1 Recruitment and Retention

**FIGURE 1:**
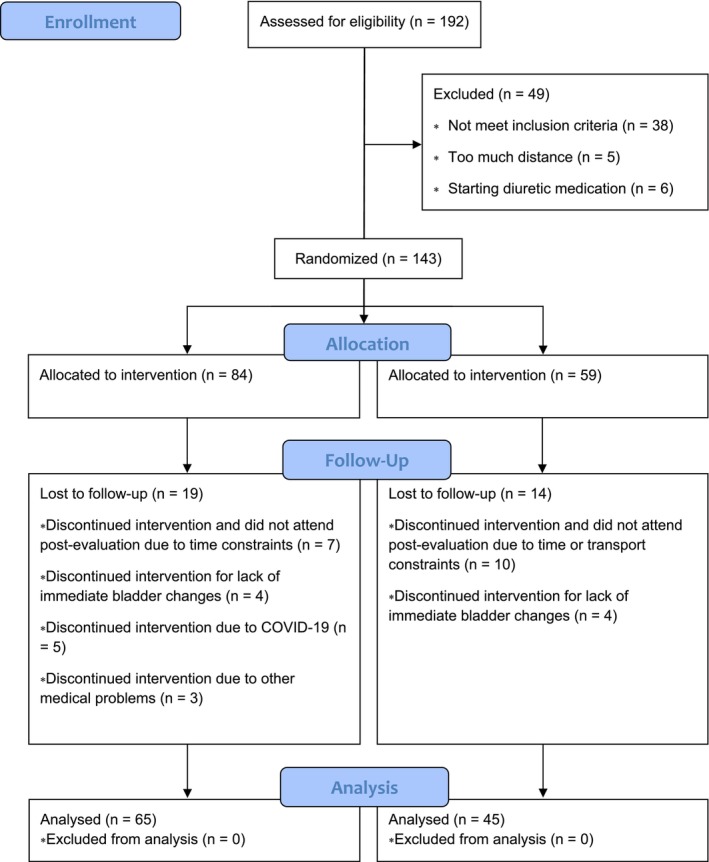
Participant recruitment and flow through the clinical trial.

A total of 402 participants were initially interested in the study; of those, 382 received the informed consent, and 114 did not fill it in and/or did not return it. Of the remaining 268 participants who completed the informed consent and sent it back, 64 did not reply via e‐mail or contact phone, and 12 preferred to participate in the future. This left 192 eligible subjects, of which 11 were lost or excluded due to distance or were starting with diuretic medication. Of the remaining 181 participants, 38 did not meet the inclusion criteria. Thus, finally, 143 participants were randomly assigned to the two groups. Nineteen subjects in SUP and 14 in PLA were lost to follow‐up. Reasons included not attending T6 due to lack of time, transport constraints, other medical problems, positive COVID‐19, lack of immediate bladder changes, or just not wanting to continue, leaving a final study population of 65 subjects in SUP and 45 subjects in PLA. The participants' flow chart is shown in Figure 1. The intervention was well tolerated, and the compliance was 76.9%.

### 3.2 Outcomes

#### 3.2.1 Participant Characteristics

The main features of the participating subjects are shown in Table 1. No significant differences were observed between groups at T0 in terms of demographics, anthropometrics, IPSS scores, or 3‐dVD variables.

**TABLE 1:** Baseline characteristics of the participants.

	SUP (*n* = 65)	PLA (*n* = 45)	*p*
Demographics			
Sex, men, *n* (%)	43 (66.2)	33 (73.3)	—
Age (years), mean (SD)	50.2 (10.87)	52.2 (11.95)	0.199
Anthropometrics, mean (SD)			
Weight (kg)	77.6 (15.32)	76.2 (16.51)	0.654
Height (cm)	170.7 (9.21)	171.7 (8.96)	0.432
Total fat mass (%)	25.3 (9.35)	24.12 (7.71)	0.473
Total fat mass (kg)	20.1 (9.74)	18.9 (8.18)	0.75
Total fat‐free mass (kg)	57.5 (10.95)	57.4 (11.29)	0.95
BMI (kg/m^2^)	26.6 (4.71)	25.5 (4.20)	0.229
BMR (kcal)	1687.9 (309.83)	1678.8 (324.15)	0.883
IPSS scores, mean (SD)			
Daytime voids (Q2)	4.18 (0.94)	3.58 (0.93)	< 0.001
Urgency (Q4)	3.09 (1)	3.09 (1.17)	0.74
Nocturia (Q7)	3.08 (0.89)	3.2 (0.89)	0.528
QoL (Q8)	5.11 (0.96)	4.82 (1.1)	0.157
Storage subscore	10.35 (1.56)	9.87 (1.47)	0.071
Total score	19.78 (4.94)	19.64 (3.98)	0.881
3‐dVD, mean (SD)			
Urgent urinary incontinence, *n* (%)	18 (27.7)	6 (13.3)	—
Daytime voids	8.67 (2.88)	9.06 (3.12)	0.605
Urgency	3 (0.76)	2.61 (0.93)	0.045
Nocturia	1.52 (1.25)	1.52 (0.84)	0.468

*Note:* Data are presented as mean (standard deviation).

Abbreviations: 3‐dVD, 3‐day voiding diary; BMI, body mass index; BMR, basal metabolic rate; IPSS, International Prostatic Symptom Score; PLA, placebo group; QoL, quality of life and SUP, supplement group.

#### 3.2.2 OAB Outcomes

**FIGURE 2:**
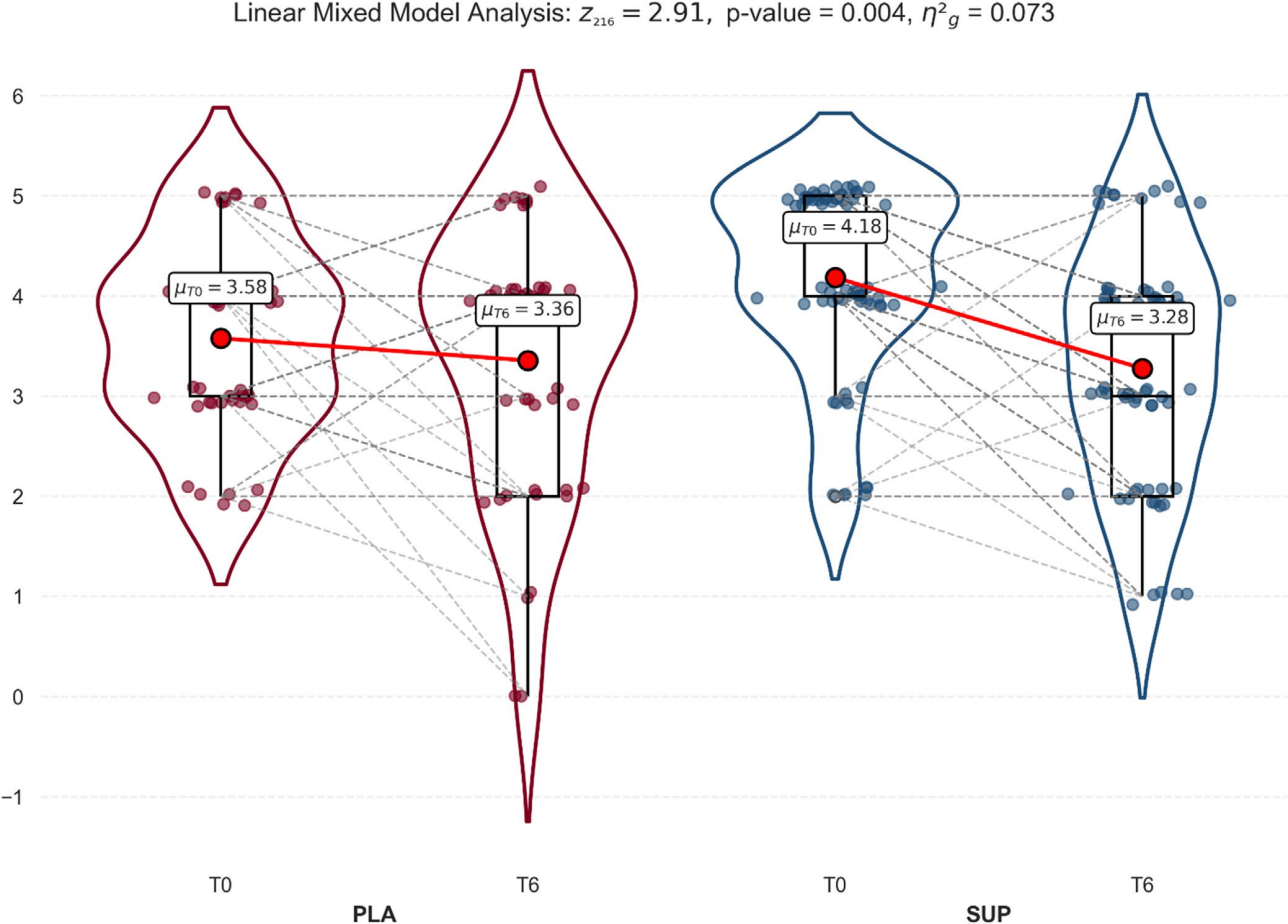
Effects of the *Angelica archangelica* supplementation on daytime voids in adults with overactive bladder. PLA: Placebo group; SUP: Supplement group. Data presented as the mean and standard deviation. Differences were assessed by a linear mixed model.

In response to the intervention, a significant effect of the interaction (group × time), with a medium effect size, was observed for daytime voids when measured by the IPSS (F_1,108_ = 8.47; *p* = 0.004; η^2^_g_ = 0.07) (Figure 2), whereas no significant differences in daytime voids were observed in the 3‐dVD. Regarding urgency, no significant differences were observed between groups for both IPSS and 3‐dVD. Finally, for nocturia, the IPSS results showed a nearly significant improvement in SUP vs PLA (F_1,108_ = 3.37; *p* = 0.069; η^2^_g_ = 0.03). The median in PLA remained at 3 from T0 to T6, while in SUP it decreased from 3 at T0 to 2 at T6. In the 3‐dVD, no significant differences were observed between groups; however, the reduction in night voids in SUP (−0.19 voids per night) was greater than the same in PLA (−0.04 voids per night) (Table 2).

**TABLE 2:** Treatment effects on overactive bladder symptomatology.

Variables	Group	T0	T6	*p*‐value for time effect	*p*‐value for group by time effect	η^2^_g_
IPSS, mean (SD)						
Daytime voids (Q2)	SUP	4.18 (0.94)	3.28 (1.16)	< 0.001	0.004	0.07
	PLA	3.58 (0.93)	3.36 (1.32)	0.362		
Urgency (Q4)	SUP	3.09 (1)	2.35 (1.12)	< 0.001	0.846	< 0.01
	PLA	3.09 (1.17)	2.4 (1.16)	0.005		
Nocturia (Q7)	SUP	3.08 (0.89)	2.34 (1.07)	< 0.001	0.069	0.03
	PLA	3.2 (0.88)	2.76 (1.04)	< 0.001		
QoL (Q8)	SUP	5.11 (0.96)	4.35 (1.32)	< 0.001	< 0.001	0.12
	PLA	4.82 (1.11)	4.71 (1.13)	0.239		
Storage subscore	SUP	10.35 (1.56)	7.97 (2.33)	< 0.001	0.025	0.05
	PLA	9.87 (1.47)	8.51 (2.48)	0.001		
Total score	SUP	19.78 (4.94)	15.89 (5.29)	< 0.001	0.495	< 0.01
	PLA	19.64 (3.98)	16.38 (5.4)	< 0.001		
3‐dVD, mean (SD)						
Daytime voids	SUP	8.67 (2.89)	8.03 (2.42)	0.035	0.745	< 0.01
	PLA	9.06 (3.12)	8.27 (2.85)	0.005		
Urgency	SUP	3 (0.76)	2.88 (0.84)	0.074	0.122	0.03
	PLA	2.61 (0.93)	2.24 (0.85)	0.002		
Nocturia	SUP	1.52 (1.25)	1.33 (0.97)	0.109	0.363	< 0.01
	PLA	1.52 (0.84)	1.48 (0.91)	0.682		

*Note:* Data are presented as mean (standard deviation).

Abbreviations: 3‐dVD, 3‐day voiding diary; IPSS, International Prostatic Symptom Score; PLA, placebo group; QoL, quality of life and SUP, supplement group.

**FIGURE 3:**
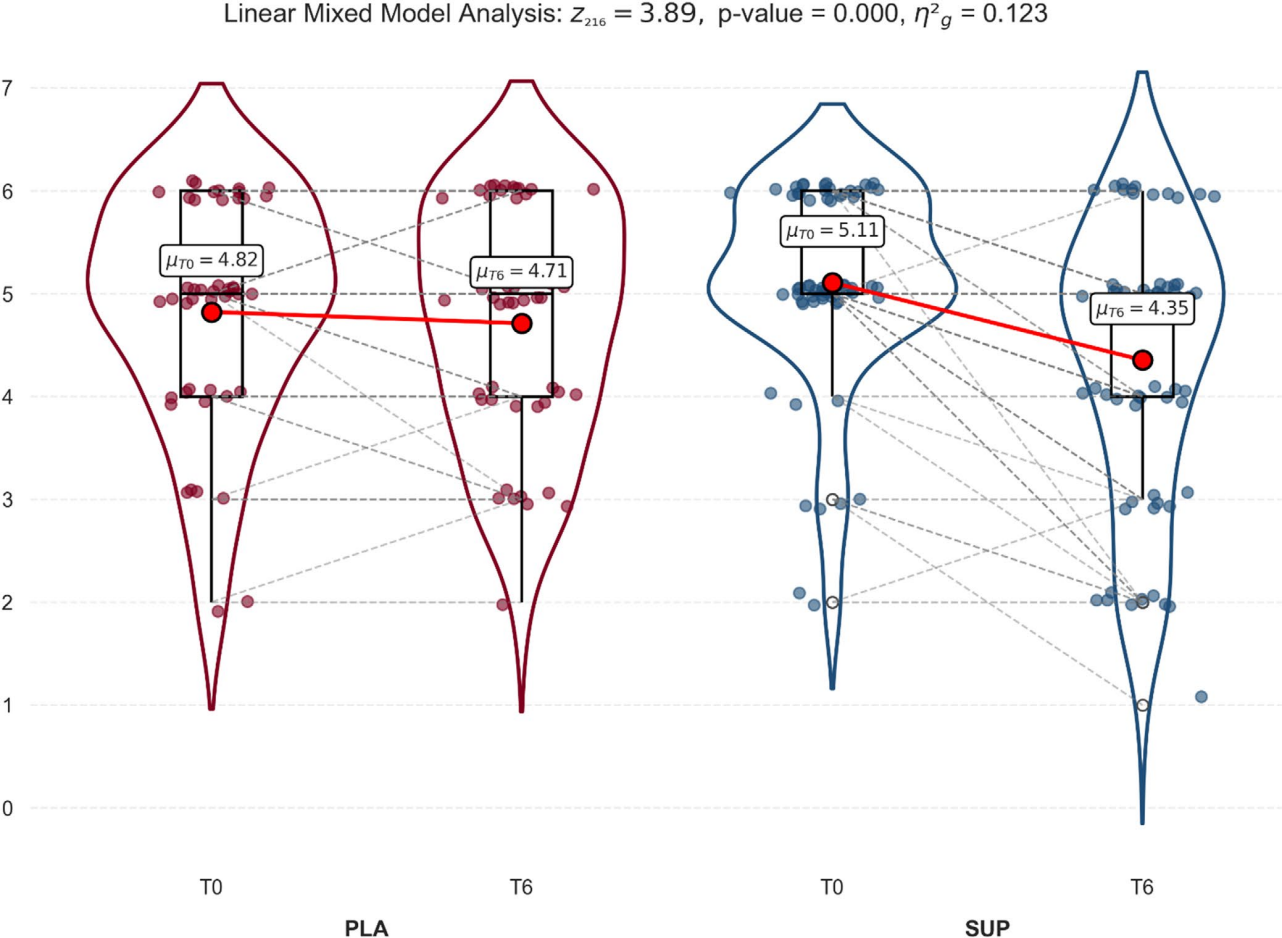
Effects of the *Angelica archangelica* supplementation on quality of life in adults with overactive bladder. PLA: Placebo group; SUP: Supplement group. Data presented as the mean and standard deviation. Differences were assessed by a linear mixed model.

Regarding QoL measured by the IPSS, results showed that SUP improved significantly compared with PLA (F_1,108_ = 15.11; *p* < 0.001; η^2^_g_ = 0.12), with a meaningful medium effect size (Figure 3).

**FIGURE 4:**
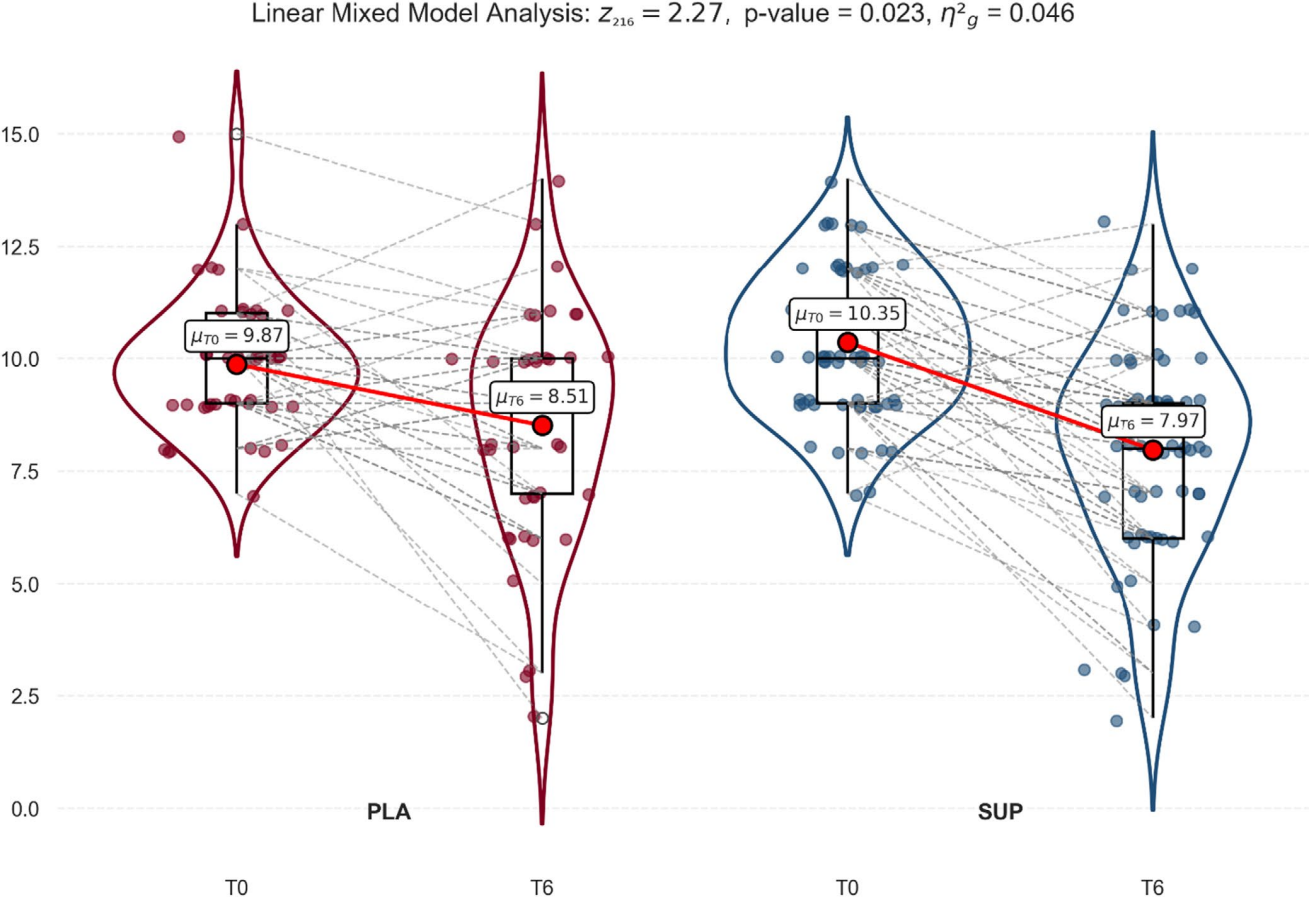
Effects of the *Angelica archangelica* supplementation on IPSS storage subscore in adults with overactive bladder. PLA: Placebo group; SUP: Supplement group. Data presented as the mean and standard deviation. Differences were assessed by a linear mixed model.

**FIGURE 5:**
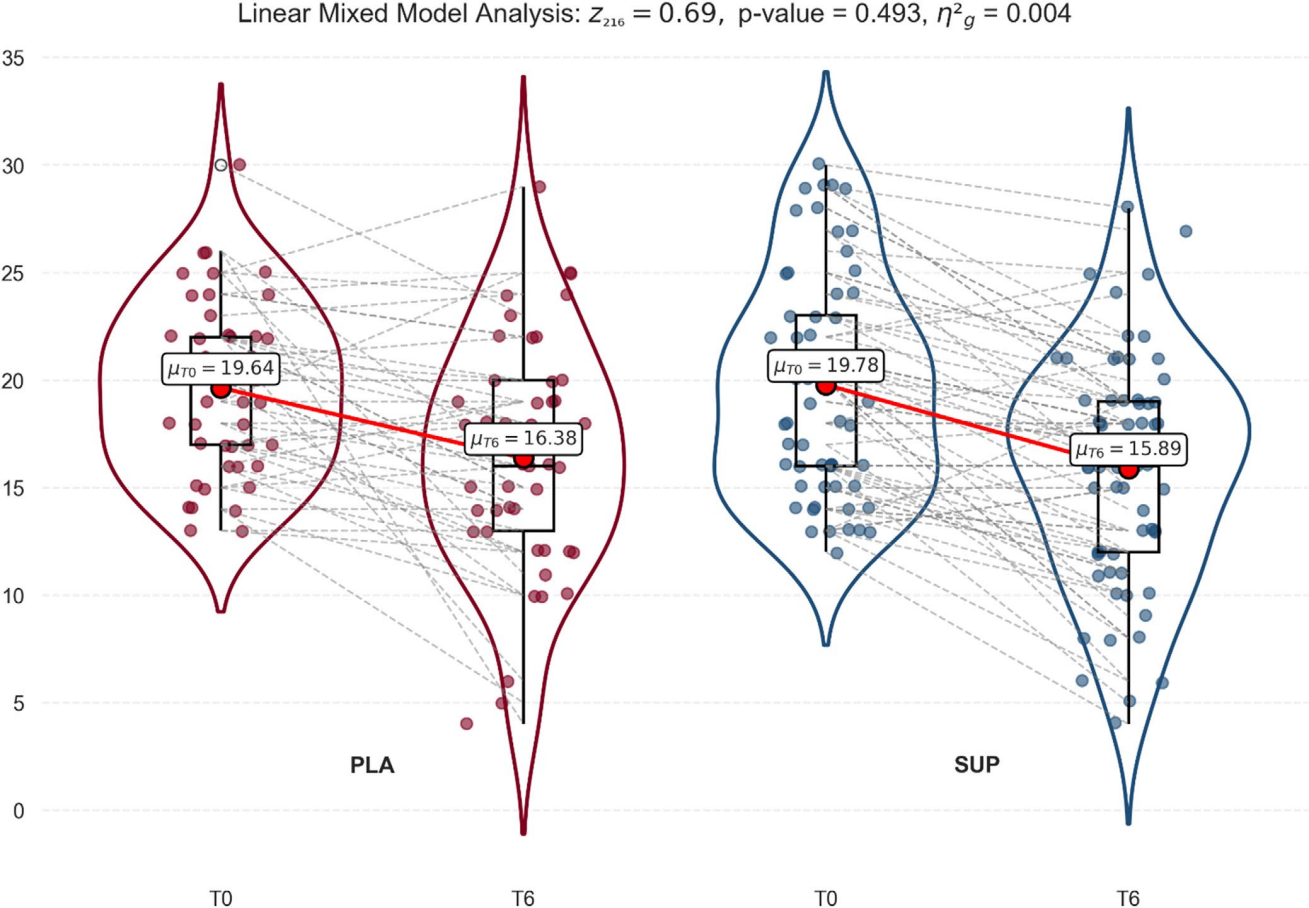
Effects of the *Angelica archangelica* supplementation on IPSS total score in adults with overactive bladder. PLA: Placebo group; SUP: Supplement group. Data presented as the mean and standard deviation. Differences were assessed by a linear mixed model.

The IPSS scores showed different results. The storage subscore improvement was significantly higher in SUP than in PLA (F_1,108_ = 5.16; *p* = 0.025; η^2^_g_ = 0.05) (Figure 4), while no differences were observed between groups for the total score. Nevertheless, regarding the IPSS total score, the SUP mean improved by nearly four points from T0 to T6, whereas PLA barely exceeded three points of improvement, suggesting that the SUP effect, in terms of clinical significance, is slightly better than PLA (Figure 5).

## 4 Discussion

This study was designed to assess the effects of a 6‐week supplement intervention with AA leaf extract in adult subjects with OAB symptoms as assessed by the IPSS and the 3‐dVD. This intervention resulted in improvements in daytime voids, QoL, and IPSS scores when a 200 mg‐AA leaf extract was administered daily for 6 weeks, highlighting the potential efficacy of this supplement in managing OAB syndrome.

OAB diagnoses rely partly on the information that subjects provide about their symptoms, the level of discomfort, and how it affects their QoL. Differential diagnosis is crucial not only to exclude other conditions but also because OAB patients may have more than one cause or diagnosis contributing to the symptoms of the lower urinary tract disease (Jiménez Cidre et al. 2016).

Although the present study did not include a specific analysis of the bioactive compounds in AA, previous research has identified the presence of coumarins (e.g., imperatorin, xanthotoxin, angelicin, bergapten) (Grabarska et al. 2020; Mahendra et al. 2020; Maurya et al. 2017; Sayed et al. 2022), essential oils rich in monoterpenes (e.g., β − phellandrene, α − pinene) and sesquiterpenes (Lowe et al. 2024), flavonoids (e.g., isoquercetin) (Di Camillo Orfali et al. 2016), and polysaccharides. These constituents have been associated with antioxidant, anti‐inflammatory, antimicrobial, and neuroactive effects. It is therefore possible that these compounds contributed to the symptomatic improvements observed in our intervention.

The significant improvement in daytime voids observed in SUP, as measured by the IPSS, suggests a beneficial effect of supplementation on this common symptom of OAB. Chapple et al. (2006) showed a decrease in daytime voids (−2.3) and nocturia (−0.6) using solifenacin, a common antimuscarinic drug, similar to those found in the present study (−1.5 and−0.66, respectively). These results also agree with those of other studies that have explored the efficacy of phytotherapeutic agents such as extracts of *Serenoa repens* in daytime voids (Vela‐Navarrete et al. 2018). However, similarly to *Serenoa repens* , the specific action of AA on detrusor overactivity or other urodynamic parameters remains unclear, as many mechanisms of action have been proposed to explain these benefits (Kumar and Bhat 2012; Seipel and Schauss 2020; Sigurdsson et al. 2013).

Urgency can be defined as “the inability to postpone urination without experiencing leakage” (Abrams et al. 2002); therefore, if a person can delay voiding without involuntary urine loss, it should not be classified as urgency. However, the various questionnaires and scales used to assess urgency have aimed to better characterize it by establishing classifications that can sometimes be confusing regarding the term and the true definition of urgency. In this study, no differences were observed for urgency symptoms, which could be due to this fact. Nevertheless, prior research on similar *Angelica* species observed its potential to reduce urgency to urinate (Chen et al. 2024).

The nearly significant improvement in nocturia symptoms found in SUP is noteworthy. Nocturia is a challenging symptom to manage and significantly impacts subjects' QoL. The RCT by Sigurdsson et al. (2013) did not show differences in nocturia between placebo and AA supplement; however, only 66 male participants were measured (Sigurdsson et al. 2013). In the current study, a slight trend toward improvement was observed among 110 participants regardless of sex. This finding suggests a potential therapeutic effect of the supplement used in the present study on nocturia symptoms. Similar effects have been reported in studies evaluating other phytotherapeutics such as isoquercitrin, a flavonoid found in AA leaf, which has been proposed to play a key role as a reducing agent in nocturia (Kowal et al. 2017). Other constituents have been found to be able to reduce the number of nocturnal voids, and the proposed mechanism of action is related to the regulation of smooth muscle receptors that control the bladder detrusor muscle activity (Chen et al. 2024). Therefore, a deeper knowledge of the main components of the supplement containing AA leaf extract is necessary to obtain clear benefits on nocturia.

Subjects with OAB primarily suffer because of the impact their symptoms have on their QoL (Abrams et al. 2000). Consequently, assessing QoL is a common feature of all self‐administered questionnaires. Then, the participant's perception of OAB symptoms ameliorating, whether they are physical or perceived changes, makes QoL a reliable and powerful indicator of subject improvement. These QoL measures are not considered in the 3‐dVD but are part of the IPSS, and therefore, the use of self‐administered questionnaires becomes essential. In this study, SUP shows a significant improvement in QoL, reinforcing the clinical relevance of the intervention. Positive changes in QoL are a critical endpoint in OAB management, as they reflect the patient‐perceived benefit of interventions. The results observed in this study are consistent with those reported using pharmacological agents such as mirabegron (Nakagomi et al. 2022) or botanical ones, such as pumpkin seed extract (Shim et al. 2014), supporting the relevance of phytotherapeutic approaches in OAB management. Given the main importance of QoL for these subjects, the results obtained in the present study are promising, indicating significant improvements in symptoms and overall well‐being. Reducing the daytime voids, urgency to urinate, and nocturia associated with OAB directly enhances physical comfort, emotional health, and social functioning (Verdejo‐Bravo et al. 2015). These outcomes demonstrate the value of comprehensive treatment approaches, highlighting their role in restoring daily functionality and addressing the psychological and social burdens of the condition.

Evaluating OAB symptoms independently is essential to understand the improvement of each one, but the overall assessment in the treatment of this syndrome and how it affects the QoL of subjects should stand out (Shawahna et al. 2021; Xu et al. 2018). At this point, the evaluation of interventions in OAB requires reliable indices to accurately assess symptom severity and treatment outcomes. The IPSS storage subscore offers valuable insights by distinguishing between voiding and storage‐related symptoms (Liao and Kuo 2014; Shalaby et al. 2013). This distinction is crucial, as storage symptoms like urgency and daytime voids are hallmarks of OAB, directly impacting QoL. The significant improvement in IPSS storage subscore in SUP highlights a potential effect specifically addressing OAB symptoms. This suggests the supplement may rebalance the dynamics between voiding and storage symptoms, contributing to overall better management of lower urinary tract symptoms (Liao and Kuo 2014). This outcome is not often reported in OAB studies but has been explored in research on urodynamic parameters. The study by Sakalis et al. (2021) found that rebalancing IPSS storage subscore correlated with reduced detrusor overactivity and improved bladder compliance. These effects could also be hypothesized for the AA supplement used in the present study due to its beneficial effects on the IPSS storage subscore. In addition, the clinical significance of the IPSS total score, with SUP nearly reaching a 4‐point change, further emphasizes its potential utility.

Despite the promising results, certain limitations of the present research must be addressed. First, to the best of our knowledge, there is no *gold standard* for the measurement of OAB symptoms, being much discrepancy about the best tools to measure them among urologists (OAB questionnaire (OAB‐q), Primary OAB Symptom Questionnaire (POSQ), OAB Symptom Score (OAB‐ss), International Consultation on Incontinence Questionnaire (ICIQ), IPSS or 3‐dVD, among others). In our case, although IPSS and 3‐dVD are among the recommended tools by the Spanish Association of Urology, many IPSS and 3‐dVD results were in the same direction, while others showed certain differences. This underlines the need for multimodal and accurate assessment tools when studying OAB. Second, although OAB subjects could have urgent urinary incontinence, urgency couldn't be statistically analyzed due to the low prevalence among the subjects studied, so in our study, urgent urinary incontinence was less relevant than the other symptoms considered. Third, the COVID‐19 pandemic hampered both the recruitment process and the follow‐up period of participants during the study. Additionally, OAB is still a taboo topic, which makes it difficult to get volunteers, resulting in a smaller sample size than we would have liked. Finally, longer follow‐up periods could provide stronger insights into the sustained efficacy of the AA supplement used.

Additionally, several strengths of our study can be highlighted. The randomized, triple‐blind, placebo‐controlled design enhances the study's quality and minimizes potential bias. Moreover, the matched pair design improves comparability between groups, reducing variability caused by confounding factors. The inclusion and exclusion criteria were rigorous, and the retention rate of the recruited sample was high, especially considering the kind of syndrome studied. Another key strength of this study is the implementation of follow‐up strategies to keep adherence throughout the intervention. The use of the RedCap platform ensured secure, centralized, and systematic data management, minimizing human errors and enhancing data integrity. Furthermore, the statistical approach was robust, incorporating linear mixed model analysis and reporting effect sizes to provide a comprehensive understanding of the results.

Future studies should also explore the active compounds in the supplement and their specific mechanisms of action. In addition, comparative trials with other botanical agents could study if AA has any synergy with alternative OAB interventions such as pelvic floor muscle training, mindfulness and relaxation techniques, or acupuncture, among others. Finally, research incorporating urodynamic testing could provide mechanistic insights, particularly regarding its effects on detrusor muscle activity and bladder compliance.

## 5 Conclusions

A 6‐week intervention with AA leaf extract was able to improve daytime voids, QoL, and IPSS storage subscore in adults with OAB symptomatology. Nocturia symptoms nearly significantly improved with the intervention vs. placebo, while the IPSS total score showed a slightly greater, though nonsignificant, improvement with the intervention compared to placebo. Urgency symptoms were not affected by the intervention.

## Author Contributions

**Jaime López‐Seoane:** conceptualization (lead), data curation (lead), formal analysis (lead), investigation (lead), methodology (lead), resources (lead), software (lead), supervision (lead), validation (lead), visualization (lead), writing – original draft (lead), writing – review and editing (lead). **Eva Gesteiro:** investigation (supporting), methodology (supporting), supervision (supporting), writing – review and editing (supporting). **María José Castro‐Alija:** investigation (supporting), writing – review and editing (supporting). **Carlos Quesada‐González:** data curation (supporting), formal analysis (supporting), software (supporting), visualization (supporting), writing – review and editing (supporting). **Margarita Pérez‐Ruiz:** supervision (supporting), writing – review and editing (supporting). **Marcela González‐Gross:** conceptualization (lead), data curation (supporting), formal analysis (supporting), funding acquisition (lead), methodology (lead), project administration (lead), resources (supporting), supervision (supporting), validation (supporting), visualization (supporting), writing – original draft (supporting), writing – review and editing (supporting).

## Funding

The study was funded through a grant agreement between the Executive Agency for Small and Medium‐sized Enterprises (EASME) of the European Commission and SagaNatura (now Florealis ehf) (agreement No. 859246 NO‐GO). Subsequently, an agreement between SagaNatura/Florealis ehf and UPM was established for the development of Project P2011600132. Further support was obtained from the Carlos III Health Institute through CIBEROBN (CB12/03/30038), co‐funded by the European Regional Development Fund. The design, management, statistical analysis, and reporting of the study were performed entirely independent of SagaNatura/Florealis ehf. The OAB study RCT was conducted by the ImFINE Research Group (gi.imfine@upm.es) of the UPM. Industries and/or private institutions had no role in the design, execution, analyses, interpretation of the data, or the decision to submit the results.

## Disclosure

The OAB study was carried out by qualified professionals for medical, clinical, food science, nutritional, and Physical Activity and Sport Sciences practice. These experts were part of the ImFINE Research Group of the Department of Health and Human Performance of the Faculty of Physical Activity and Sport Sciences of the UPM.

## Ethics Statement

Institutional Review Board Statement: This research was conducted in accordance with the Ethical Guidelines of the Declaration of Helsinki of 1964 and further amendments (“Declaration of Helsinki. Recommendations Guiding Doctors in Clinical Research,” 1964; World Medical Association 2013) and following the Spanish and European regulations on data protection. The protocol was approved by the Bioethics Committee of the UPM (reference number 20200305–1, date of approval 16/03/2020) for studies involving humans.

## Consent

Informed consent was obtained from all subjects involved in the study before their participation.

## Conflicts of Interest

The authors declare no conflicts of interest.

## Supporting information


**Figure S1:** Mean differences between baseline (T0) and week 6 (T6) for each IPSS variable and compared by group. PLA: placebo group; SUP: supplement group. * = significant differences between PLA and SUP assessed by Linear Mixed Model Analysis.

## Data Availability

The data that support the findings of this study are available from the corresponding author upon reasonable request.
