# Supplementary material for: Effects of Angelica archangelica Extract on Overactive Bladder: A Pilot Randomized Controlled Trial
Source: Food Sci Nutr. 2025 Dec 8;13(12):e71258. doi: 10.1002/fsn3.71258 (PMC12683295; doi:10.1002/fsn3.71258)
Supplement: Supplementary file 1 — Figure S1: Mean differences between baseline (T0) and week 6 (T6) for each IPSS variable and compared by group. PLA: placebo group; SUP: supplement group. * = significant differences between PLA and SUP assessed by Linear Mixed Model Analysis. [file FSN3-13-e71258-s001.docx]

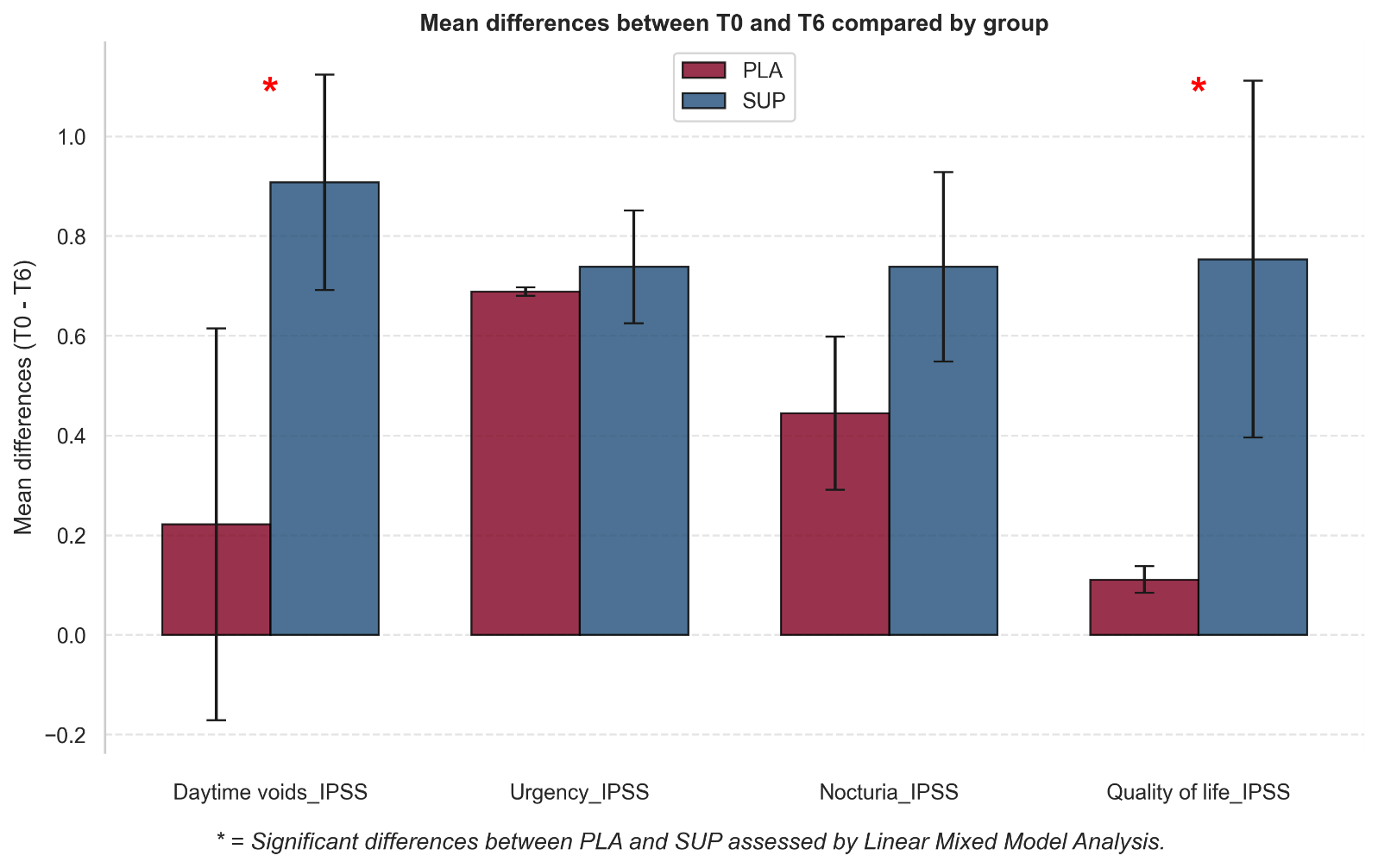


**Figure S1.** Mean differences between baseline (T0) and week 6 (T6) for each IPSS variable and compared by group. PLA: placebo group; SUP: supplement group. * = significant differences between PLA and SUP assessed by Linear Mixed Model Analysis.

**Figure S1**
